# Effect of evidence-based nursing supervision on hemodialysis patients with chronic kidney failure

**DOI:** 10.1097/MD.0000000000044777

**Published:** 2025-10-10

**Authors:** Wen Ren, Zhiyu Chen, Xiaolan Liu, Xueli Zhu

**Affiliations:** a Department of Nephrology, West China Hospital, Sichuan University/West China School of Nursing, Sichuan University, Chengdu, Sichuan Province, China.

**Keywords:** chronic renal failure, evidence-based supervision, hemodialysis, high-quality nursing management, nursing effect

## Abstract

This study aims to evaluate the effects of an evidence-based supervision nursing model on self-management, quality of life, emotional well-being, and complication rates in patients with chronic renal failure (CRF) undergoing hemodialysis. A retrospective cohort analysis was conducted on 160 patients with CRF receiving hemodialysis between January 2022 and December 2023. After minimizing potential biases, patients were randomly assigned to either Group A (n = 80), who received evidence-based supervision nursing under a high-quality management model, or Group B (n = 80), who received traditional nursing care. Key outcomes, including self-management skills, quality of life (as measured by the Short Form Health Survey), emotional burden, and incidence of complications, were compared between the 2 groups. Patients in Group A demonstrated significantly improved self-management abilities, particularly in peer relationships, problem-solving, self-care practices, and emotional regulation, compared to Group B (*P* < .01). Group A also showed significantly higher scores on the Short Form Health Survey quality of life assessment (*P* < .01, *P* < .05). Furthermore, Group A reported lower perceived emotional burdens and exhibited reduced anxiety and depression scores (as measured by Self-Rating Anxiety Scale and Self-Rating Depression Scale) compared to Group B (*P* < .01). In addition, the incidence of complications, such as catheter thrombosis, vascular stenosis, and aneurysms, was significantly lower in Group A (*P* < .01, *P* < .05). Implementing an evidence-based supervision nursing model in patients with CRF undergoing hemodialysis significantly improves self-management capabilities, enhances quality of life, and reduces emotional burden and the risk of complications. This approach offers a superior alternative to traditional nursing care for these patients.

## 1. Introduction

Chronic renal failure (CRF) represents the advanced stage of various kidney diseases, characterized by progressive atrophy of renal tissues and accompanied by symptoms such as nausea, vomiting, anemia, as well as severe water, electrolyte, and acid–base disturbances. As kidney function deteriorates irreversibly over time, multiple organ systems may be affected, substantially impairing patients’ daily life quality.^[1]^

Hemodialysis is currently the standard treatment for CRF, operating through extracorporeal circulation to eliminate metabolic wastes and maintain internal equilibrium.^[2–4]^ Nevertheless, the necessity for prolonged and repeated dialysis often imposes considerable physical exhaustion, psychological distress, and financial hardship on patients, further exacerbated by the chronicity of their primary renal condition.^[5–7]^

Traditional nursing interventions predominantly address physical care during dialysis sessions but often fall short in providing comprehensive psychological support, individualized education, and guidance in self-management. Such limitations have been linked to reduced treatment compliance, poorer emotional resilience, and an elevated risk of complications among dialysis patients.^[8]^ This indicates an urgent need for more holistic, patient-centered nursing approaches in the management of chronic renal failure (CRF).

Evidence-Based Nursing has been increasingly advocated as an effective strategy to bridge these gaps, integrating clinical expertise, the latest scientific evidence, and patients’ values into the care process.^[9,10]^ In particular, the incorporation of evidence-based supervision into Evidence-Based Nursing frameworks enhances adherence to standardized care protocols by promoting continuous evaluation, targeted interventions, and active patient engagement.^[11–13]^ This supervision not only ensures the fidelity of evidence application in practice but also dynamically adjusts care according to patient feedback and clinical changes, thus optimizing overall outcomes.

In light of the complex needs of CRF patients undergoing hemodialysis, this study aims to evaluate the effectiveness of an evidence-based supervision nursing model, focusing on improvements in self-management abilities, quality of life, emotional health, and the incidence of dialysis-related complications. The findings are expected to provide a robust foundation for advancing high-quality nursing management in clinical nephrology practice.

## 2. Materials and methods

### 2.1. Clinical data

**Figure 1.**
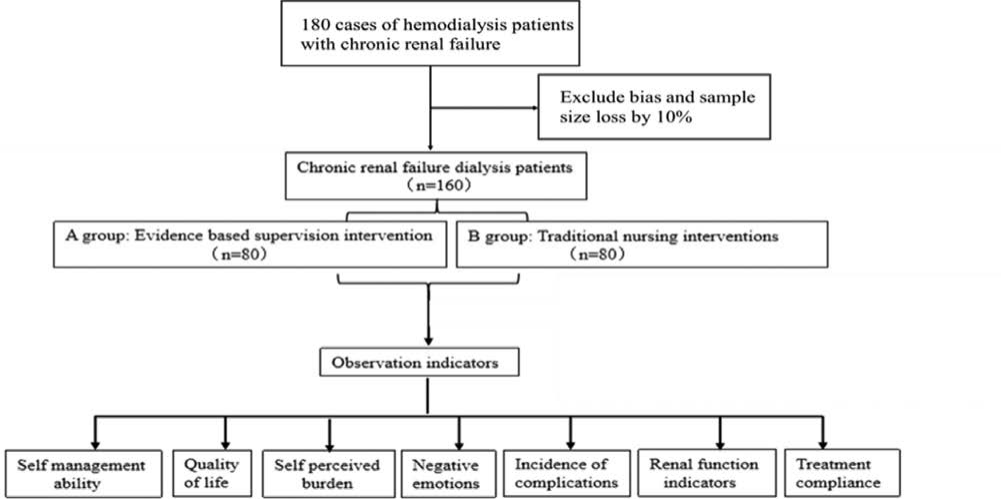
Flow chart.

This study was a retrospective cohort study involving 160 patients who had already received hemodialysis care interventions within a specified time frame (January 2022–December 2023). Data was collected retrospectively from medical records and patients were classified according to the type of care they received. Patients in Group A received evidence-based nursing care, and patients in Group B received traditional nursing care. As shown in Figure 1.

Inclusion criteria were: meeting the diagnostic criteria for CRF and confirmed diagnosis; meeting the indications for hemodialysis; having a relatively stable condition; providing informed consent and cooperating with the study. The exclusion criteria were: presence of other malignant tumors; severe organic lesions of vital organs such as the heart, liver, or kidneys; mental illness or significant cognitive impairment; blood disorders or coagulation dysfunction. The patients were randomly divided into Group A and Group B, with 90 cases in each group. Group A included 46 males and 44 females, with an average age of 58.45 ± 4.27 years and an average disease course of 5.21 ± 0.18 years. Primary diseases included chronic pyelonephritis (24 cases), glomerulonephritis (44 cases), renal arteriolar sclerosis (10 cases), and diabetic nephropathy (2 cases). The average duration of hemodialysis was 1.28 ± 0.13 years. Catheter placement was on the left side in 50 cases and on the right side in 40 cases. Educational levels were junior high school (16 cases), high school or technical secondary school (40 cases), and college or above (34 cases).

Group B included 44 males and 46 females, with an average age of 57.81 ± 4.35 years and an average disease course of 5.17 ± 0.22 years. Primary diseases included chronic pyelonephritis (30 cases), glomerulonephritis (48 cases), renal arteriolar sclerosis (8 cases), and diabetic nephropathy (4 cases). The average duration of hemodialysis was 1.31 ± 0.11 years. Catheter placement was on the left side in 46 cases and on the right side in 44 cases. Educational levels were junior high school (12 cases), high school or technical secondary school (46 cases), and college or above (32 cases). There were no statistically significant differences in gender, age, disease course, and other general data between the 2 groups (*P *> .05). This study complies with the relevant ethical principles outlined in the Declaration of Helsinki.

### 2.2. Methods

#### 2.2.1. Sample size calculation

All sample data in this study were analyzed retrospectively. The research subjects were 180 CRF hemodialysis patients admitted from January 2022 to December 2023. Divide into 2 groups, A and B, based on different nursing methods. This study used SPSS 22.0 statistical software (IBM, Armonk) to complete the statistical analysis, and was divided into statistical groups in a 1:1 ratio, with 80 cases in each group. Considering the risk of bias and the existence of a 10% sample loss rate, each group had at least 90 people.

#### 2.2.2. Grouping method

Data were retrospectively collected from medical records, and patients were categorized based on the type of nursing care they received. Group A received evidence-based supervision nursing, while Group B received traditional nursing care. While patients were categorized based on the care they had received historically, this study retrospectively evaluated outcomes such as self-management, quality of life, and complication rates in these predefined groups. No new interventions were introduced during the study period.

#### 2.2.3. Group B

Group B received conventional hemodialysis nursing care, detailed as follows. Upon entering the ward and completing the admission process, the responsible nurse provided the patients with a thorough introduction to the department and ward environment, alleviating their discomfort and unfamiliarity. Health education was conducted using pamphlets or videos to help patients understand their condition and enhance their knowledge of hemodialysis. Based on this, the responsible nurse could better understand the patients’ psychological status and systematically address any negative emotions. The nurse enhanced communication with the patients, promptly answered disease-related questions, regularly patrolled, monitored patient conditions, and promptly managed any emergencies.

#### 2.2.4. Group A

Group A received interventions based on the evidence-based supervision method under a high-quality nursing management model, detailed as follows:

1. Establishing an evidence-based supervision team: forming a supervisory team comprising nurses with extensive experience in nephrology and chief nursing officers. They undergo regular training in nephrology knowledge related to hemodialysis, conducted once a week. Simultaneously, they study the theory and skills of evidence-based supervisory nursing. Upon completion of training, timely assessments of the team members are conducted on the training content. Only those who pass the assessment are eligible to participate in the team’s nursing activities.
2. Conducting lectures on chronic renal failure and hemodialysis knowledge: based on the patients’ education levels, targeted explanations on hemodialysis treatment were provided, with an emphasis on possible adverse situations during hemodialysis. Patients were encouraged to actively participate and ask questions about their condition, thereby enhancing their understanding of the disease. Intervention attendance and frequency of instruction: patients in the intervention group attended weekly education lectures on CRF and hemodialysis management. Attendance was monitored, and the average attendance per patient was 80%. During each hemodialysis session, caregivers provided personalized instructions to patients, averaging 6 instructions per patient throughout the study period. This ensures consistent patient engagement and adherence to evidence-based surveillance protocols.
3. Addressing patients’ emotional well-being: due to the prolonged nature of hemodialysis and renal failure, patients frequently experience negative emotions such as depression and anxiety, impacting their mental health. The nursing team engaged in active communication with patients to alleviate these emotions and promoted peer support to encourage mutual encouragement and build confidence in their treatment.
4. Supervising hemodialysis nursing care: prior to hemodialysis, patients’ psychological states were assessed to understand and reduce their emotional burden. Deep breathing exercises and other stress-relief techniques were guided. During hemodialysis, 35°C dialysis fluid was used, and patients were monitored to refrain from eating, except for specific high-protein diets if necessary. The nursing team observed vital signs closely, with increased rounds to detect any abnormalities, promptly reporting to physicians for management. After treatment, patients were supervised in controlling their diet, particularly protein and sodium intake, to prevent complications. Additionally, they were encouraged to engage in suitable aerobic exercises.

#### 2.3.5. Observation indicators

1. Self-management ability: 1 month following dialysis treatment, both groups’ self-management skills were evaluated using a standardized self-management scale.^[14,15]^ The scale measures 4 key areas: problem-solving skills, peer relationships, emotional regulation, and self-care execution, with responses scored on a 4-point scale. Higher scores correspond to improved self-management abilities.
2. Quality of life: post-intervention, quality of life in both groups was assessed using the Short Form Health Survey (SF-36), which evaluates 4 domains: material well-being, social functioning, physical health, and psychological status. Each domain is scored up to a maximum of 100 points, with higher total scores indicating a better quality of life.
3. Perceived burden: before and after the intervention, the perceived burden scale was used to score the economic, emotional, and physical burdens of both groups. Higher scores indicate a heavier perceived burden by the patients. The Zarit Burden Interview is a widely used scale that evaluates the subjective burden experienced by individuals, where higher scores indicate a greater perceived burden. Although originally developed for caregivers, the Zarit Burden Interview has been adapted to assess perceived burden in patients with chronic illness, capturing multiple dimensions of their economic, emotional, and physical burdens.
4. Negative emotions: the Self-Rating Anxiety Scale (SAS) and Self-Rating Depression Scale (SDS) were used to assess anxiety and depression before and after the intervention. An SAS score > 50 indicates anxiety, and an SDS score > 53 indicates depression. Higher scores correspond to more severe anxiety and depression.
5. Incidence of complications: the occurrence of vascular stenosis, aneurysms, and catheter thrombosis was recorded and compared between the 2 groups.
6. Renal function indicators: peripheral venous blood (3 mL) was collected before and after the intervention. Serum creatinine (Scr) and blood urea nitrogen (BUN) levels were measured using the double-antibody sandwich enzyme-linked immunosorbent assay. The samples were processed following standard procedures, and absorbance was measured at 450 nm using a Bio-Rad microplate reader (Hercules).
7. Treatment adherence: adherence was assessed based on patients’ participation in hemodialysis, medication compliance, and adherence to post-discharge advice. Complete adherence was defined as full participation and behavior change; partial adherence as completing dialysis with some behavior change; non-adherence as lack of cooperation and no behavior change. Total adherence rate = complete + partial adherence rate.

### 2.4. Statistical methods

Data were analyzed retrospectively to assess the effectiveness of the evidence-based supervision model by comparing the outcomes of patients already exposed to different nursing approaches. Data were processed using SPSS 22.0 statistical software. Categorical data were expressed as numbers and percentages and analyzed using the Chi-square (χ²) test. Continuous data following a normal distribution were expressed as mean ± standard deviation ($\bar{x}\pm s$) and analyzed using the *t* test. *P* < .05 was considered statistically significant.

## 3. Results

### 3.1. Comparison of self-management abilities between the 2 groups after intervention

After the intervention, Group A demonstrated significantly higher scores than Group B in all 4 aspects of self-management: peer relationships, problem-solving, self-care execution, and emotional handling. Specifically, Group A’s scores were 13.56 ± 4.37, 18.56 ± 3.56, 19.89 ± 5.65, and 13.74 ± 3.01, respectively, compared to 11.89 ± 2.23, 14.64 ± 2.13, 15.16 ± 3.75, and 10.45 ± 1.42 for Group B. These differences were statistically significant (*P* < .05) (see Table 1).

**Table 1:** Comparison of self-management ability between 2 groups after intervention (score, $\bar{x}\pm \;s$).

Group	n	Peer relationships	Problem-solving ability	Execution of self-care	Emotional handling
A group	80	13.56 ± 4.37	18.56 ± 3.56	19.89 ± 5.65	13.74 ± 3.01
B group	80	11.89 ± 2.23	14.64 ± 2.13	15.16 ± 3.75	10.45 ± 1.42
*t*		3.049	5.976	4.412	6.252
*P*		.003	<.001	<.001	<.001

### 3.2. Comparison of SF-36 scores between the 2 groups after intervention

Following the intervention, Group A showed significantly higher SF-36 scores than Group B in several domains. Specifically, the material life, psychological state, physical health, and social function scores were all significantly higher in Group A compared to Group B. These differences were statistically significant (*P* < .05) (see Table 2).

**Table 2:** Comparison of SF-36 scores between 2 groups after intervention (score, $\bar{x}\pm \;s$).

Group	n	Material life	Psychological state	Physical health	Social function
A group	80	86.13 ± 4.37	88.26 ± 5.48	86.59 ± 4.17	84.57 ± 3.29
B group	80	84.28 ± 3.17	82.15 ± 3.47	81.14 ± 3.26	82.64 ± 3.28
*t*		2.167	5.958	6.512	2.628
*P*		.003	<.001	<.001	.010

SF-36 = Short Form Health Survey.

### 3.3. Comparison of perceived burden between the 2 groups before and after intervention

Before the intervention, no significant differences were found in emotional, economic, and physical burdens between the 2 groups (*P* > .05). After the intervention, Group A exhibited significantly lower emotional, economic, and physical burden scores compared to Group B, with all scores being significantly reduced in Group A (*P* < .05) (see Table 3).

**Table 3:** Comparison of self-perceived burden between 2 groups before and after intervention (score, $\bar{x}\pm \;s$).

Group	n	Emotional burden		Financial burden		Physical burden	
		Before intervention	After intervention	Before intervention	After intervention	Before intervention	After intervention
A group	80	3.67 ± 0.12	2.14 ± 0.24	14.97 ± 1.14	7.34 ± 1.64	15.84 ± 2.65	7.84 ± 2.51
B group	80	3.74 ± 0.34	3.21 ± 0.55	15.64 ± 2.41	10.14 ± 1.46	16.12 ± 1.45	11.23 ± 2.14
*t*		1.228	11.277	1.589	8.065	0.586	6.501
*P*		.223	<.001	.116	<.001	.559	<.001

### 3.4. Comparison of SAS and SDS scores between the 2 groups before and after intervention

Before the intervention, no significant differences were found in SAS and SDS scores between the 2 groups (*P* > .05). After the intervention, Group A exhibited significantly lower SAS and SDS scores compared to Group B, indicating reduced anxiety and depression. These differences were statistically significant (*P* < .05) (see Table 4).

**Table 4:** Comparison of SAS and SDS scores between 2 groups before and after intervention (score, $\bar{x}\pm \;s$).

Group	n	SAS score		SDS score	
		Before intervention	After intervention	Before intervention	After intervention
A group	80	58.74 ± 5.44	37.45 ± 5.74	64.12 ± 4.89	42.33 ± 4.78
B group	80	57.54 ± 6.14	44.66 ± 4.78	63.41 ± 5.12	46.21 ± 6.56
*t*		0.925	6.105	0.634	3.004
*P*		.357	<.001	.528	.004

SAS = Self-Rating Anxiety Scale, SDS = Self-Rating Depression Scale.

### 3.5. Comparison of treatment adherence between the 2 groups

After the intervention, Group A demonstrated significantly higher overall treatment adherence compared to Group B, with 95.00% of patients in Group A adhering to treatment, compared to 76.25% in Group B (*P* = .013). Additionally, Group A had higher rates of full and partial adherence and significantly lower rates of non-adherence (see Table 5).

**Table 5:** Comparison of treatment compliance between 2 groups of patients (n = 80, n/%).

Group	Full adherence	Partial adherence	Non-adherence	Total adherence
A group	36/45.00	40/50.00	4/5.00	95.00
B group	25/31.25	36/45.00	19/23.75	76.25
χ^2^*/P*				6.222/.013

### 3.6. Comparison of renal function indicators before and after intervention in both groups

Before the intervention, there were no significant differences in Scr and BUN levels between the 2 groups (*P* > .05). After the intervention, both Scr and BUN levels significantly decreased in both groups, with Group A showing lower levels than Group B (*P* < .05) (see Table 6).

**Table 6:** Comparison of renal function indicators before and after intervention between 2 groups of patients (n = 80, $\bar{x}\pm \;s$).

Group	Scr (μmol/L)		BUN (mmol/L)	
	Before intervention	After intervention	Before intervention	After intervention
A group	814.29 ± 65.11	412.35 ± 37.10*	41.30 ± 4.19	19.23 ± 2.53*
B group	810.55 ± 63.59	511.12 ± 50.07*	41.15 ± 4.07	33.40 ± 5.00*
*t*/*P*	0.266 ± 0.791	10.270/.000	0.166/.868	16.390/.000

BUN = blood urea nitrogen, Scr = serum creatinine.

*P < .05.

*Note*: compared with before intervention in the same group, *P < .05.

### 3.7. Comparison of occurrence of complications after intervention between the 2 groups

After the intervention, the rates of catheter thrombosis were 2.50% in Group A and 20.00% in Group B, with a significant difference between the 2 groups (*P* = .003 < 0.05). The rates of vascular stenosis were 7.50% in Group A and 25.00% in Group B, also showing a significant difference (*P* = .034 < .05). The rates of aneurysm were 5.00% in Group A and 22.50% in Group B, with a significant difference (*P* = .045 < .05) between the 2 groups. These results indicate that the occurrence of complications in Group A was lower than that in Group B (see Table 7).

**Table 7:** Comparison of incidence of related complications between 2 intervention groups.

Group	n	Catheter thrombosis		Vascular stenosis		Aneurysm	
		Cases	%	Cases	%	Cases	%
A group	80	2	2.50	6	7.50	4	5.00
B group	80	16	20.00	20	25.00	18	22.50
χ^2^		4.070		4.501		4.011	
*P*		.003		.034		.045	

## 4. Discussion

CRF results in the accumulation of toxins in the body, which can lead to life-threatening complications if not effectively managed. Currently, hemodialysis is the primary treatment for patients with CRF, providing a means to filter waste and maintain fluid balance.^[16–18]^ However, prolonged dialysis often leads to psychological distress, including anxiety and depression, which, if not addressed, can negatively impact the treatment outcomes, such as blood pressure and heart rate regulation, ultimately affecting dialysis efficacy.^[19]^

Our study focused on improving the care quality for hemodialysis patients using an evidence-based nursing supervision model, which has been shown to enhance patient outcomes by addressing both physical and emotional needs. The results of this study demonstrate significant improvements in self-management and quality of life among patients in Group A, who received evidence-based supervision. Group A showed significantly higher self-management scores and SF-36 quality of life scores compared to Group B (*P* < .01, *P* < .05), reflecting the positive impact of tailored nursing interventions that focus on enhancing self-care practices, emotional regulation, and problem-solving abilities. These improvements are consistent with previous studies emphasizing the importance of patient engagement and self-management in chronic disease management.^[20–23]^

By fostering effective communication between healthcare providers and patients, Group A patients were empowered to take an active role in their care. This not only boosted their self-efficacy but also reinforced their confidence in managing their condition. As a result, they exhibited lower perceived burdens and reduced levels of anxiety and depression, as indicated by significantly lower SAS and SDS scores (*P* < .01) compared to Group B. These findings align with the well-established link between psychological well-being and adherence to medical treatment in chronic disease patients.^[24]^ Moreover, Group A’s higher treatment adherence rate (95% vs 76.25%, *P* = .013) suggests that integrating psychological support into nursing care can significantly improve patient compliance, further highlighting the importance of a holistic approach to patient management.

In addition to improving emotional well-being and treatment adherence, our results also demonstrated a significant reduction in the incidence of dialysis-related complications in Group A. Post-intervention, Group A exhibited significantly lower rates of catheter thrombosis, vascular stenosis, and aneurysms compared to Group B (*P* < .05). This improvement can be attributed to the increased frequency of monitoring and timely interventions facilitated by the evidence-based supervisory model. The higher level of supervision and monitoring allowed for better management of complications, which is consistent with prior research suggesting that structured nursing supervision reduces adverse events in hemodialysis patients.^[25,26]^

While these findings are promising, the study has certain limitations. The relatively small sample size may introduce some bias, affecting the generalizability of the results. Additionally, the study focused on a limited set of observation indicators, and future studies should incorporate a broader range of clinical outcomes and psychological measures to provide more comprehensive insights into the long-term effects of evidence-based supervision in hemodialysis care.

To address these limitations, we plan to expand the sample size and incorporate additional indicators, such as renal function biomarkers and long-term psychological outcomes. This will provide a more robust dataset, further validating the benefits of the evidence-based nursing model for chronic kidney disease management.

## 5. Conclusion

In summary, under the high-quality nursing model, evidence-based supervision is more effective, and the self-management ability and quality of life of patients with chronic kidney failure undergoing hemodialysis are not only effectively improved, but also their self-perceived burden, negative emotion score, and incidence of related complications are significantly reduced.

## Author contributions

**Conceptualization:** Wen Ren, Zhiyu Chen, Xiaolan Liu, Xueli Zhu.

**Data curation:** Wen Ren, Xiaolan Liu.

**Formal analysis:** Wen Ren, Xiaolan Liu.

**Investigation:** Wen Ren, Zhiyu Chen, Xueli Zhu.

**Methodology:** Wen Ren, Zhiyu Chen, Xueli Zhu.

**Supervision:** Zhiyu Chen.

**Validation:** Wen Ren, Zhiyu Chen.

**Writing – original draft:** Wen Ren, Xueli Zhu.

**Writing – review & editing:** Wen Ren, Xueli Zhu.

## References

[1] Olsen E van Galen G. Chronic renal failure-causes, clinical findings, treatments and prognosis. Vet Clin North Am Equine Pract. 2022;38:25–46. 35365250 10.1016/j.cveq.2021.11.003

[2] Terzo C Gembillo G Cernaro V. Investigational new drugs for the treatment of chronic renal failure: an overview of the literature. Expert Opin Investig Drugs. 2024;33:319–34. 10.1080/13543784.2024.2326624 38429874

[3] van Tonder CB Joubert G Moodley A. Restless legs syndrome in chronic renal failure patients on dialysis. Afr Health Sci. 2023;23:764–77. 10.4314/ahs.v23i3.88 PMC10862641 38357105

[4] Zhu X Han Q Xia L Shang J Yan X. Efficacy of two hemodialyses in patients with chronic renal failure complicated by massive intracerebral hemorrhage. Ann Clin Transl Neurol. 2023;10:1186–99. 37350299 10.1002/acn3.51800 PMC10351664

[5] Lopez T Banerjee D. Management of fluid overload in hemodialysis patients. Kidney Int. 2021;100:1170–3. 34802558 10.1016/j.kint.2021.09.013

[6] Kraus MA Fluck RJ Weinhandl ED. Intensive hemodialysis and health-related quality of life. Am J Kidney Dis. 2016;68(5s1):S33–42. 27772641 10.1053/j.ajkd.2016.05.023

[7] Alencar SBV de Lima FM Dias LDA. Depression and quality of life in older adults on hemodialysis. Braz J Psychiatry. 2020;42:195–200. 31389496 10.1590/1516-4446-2018-0345 PMC7115449

[8] Weng Y Hu L Zhuang S Shi Y Xu C Liu C. Effects of high-flux hemodialysis with narrative care on clinical efficacy and prognostic quality of life of patients with chronic renal failure. Altern Ther Health Med. 2023;29:164–9. 36933248

[9] Ramos EC Santos Ida S Zanini Rde V Ramos JM. Quality of life of chronic renal patients in peritoneal dialysis and hemodialysis. J Bras Nefrol. 2015;37:297–305. 26398639 10.5935/0101-2800.20150049

[10] Price L Osborne S. CPD: using evidence based clinical practice guidelines in the management of anaemia in patients with chronic renal failure. Collegian. 2003;10:35–7. 15481510 10.1016/s1322-7696(08)60619-8

[11] Ha Dinh TT Bonner A Clark R Ramsbotham J Hines S. The effectiveness of the teach-back method on adherence and self-management in health education for people with chronic disease: a systematic review. JBI Database System Rev Implement Rep. 2016;14:210–47. 10.11124/jbisrir-2016-2296 26878928

[12] Neoh K Page A Chin-Yee N Doree C Bennett MI. Practice review: evidence-based and effective management of anaemia in palliative care patients. Palliat Med. 2022;36:783–94. 35331051 10.1177/02692163221081967 PMC9087312

[13] Zarit SH Reever KE Bach-Peterson J. Relatives of the impaired elderly: correlates of feelings of burden. Gerontologist. 1980;20:649–55. 7203086 10.1093/geront/20.6.649

[14] Dai Z Jing S Liu X. Development and validation of the diabetic self-management scale based on information–motivation–behavioral skills theory. Front Public Health. 2023;11:1109158. 36908406 10.3389/fpubh.2023.1109158 PMC9998917

[15] Kim ES. Development and effect of a rational–emotive–behaviour-therapy-based self-management programme for early renal dialysis patients. J Clin Nurs. 2018;27:4179–91. 29968272 10.1111/jocn.14608

[16] Kurtz C Geron R Shadmi E. Interest and perceived capability of self-care in haemodialysis units. J Clin Nurs. 2021;30:645–54. 33289199 10.1111/jocn.15584

[17] Palevsky PM. Dialysis modality and dosing strategy in acute renal failure. Semin Dial. 2006;19:165–70. 16551296 10.1111/j.1525-139X.2006.00144.x

[18] Ronco C. Hemodiafiltration: technical and clinical issues. Blood Purif. 2015;40(Suppl 1):2–11. 26344507 10.1159/000437403

[19] Song MK Ward SE Hladik GA Bridgman JC Gilet CA. Depressive symptom severity, contributing factors, and self-management among chronic dialysis patients. Hemodial Int. 2016;20:286–92. 25998623 10.1111/hdi.12317 PMC4654980

[20] Hsu SH Lin YL Koo M Creedy DK Tsao Y. Health-literacy, self-efficacy and health-outcomes of patients undergoing haemodialysis: mediating role of self-management. J Ren Care. 2024;50:342–52. 38522017 10.1111/jorc.12493

[21] Curtin RB Sitter DC Schatell D Chewning BA. Self-management, knowledge, and functioning and well-being of patients on hemodialysis. Nephrol Nurs J. 2004;31:378–86, 396; quiz 387. 15453230

[22] Fiset VJ Graham ID Davies BL. Evidence-based practice in clinical nursing education: a scoping review. J Nurs Educ. 2017;56:534–41. 28876439 10.3928/01484834-20170817-04

[23] Zhao J Liu X Zhang W Xing Y Cho SW Hao Y. Evidence-based nursing outputs and hot spot analysis of the last 5 years in mainland China: results of a bibliometric analysis. Int J Nurs Pract. 2018;24:e12628. 29498139 10.1111/ijn.12628

[24] Crawford CL Rondinelli J Zuniga S. Testing of the nursing evidence-based practice survey. Worldviews Evid Based Nurs. 2020;17:118–28. 32233058 10.1111/wvn.12432

[25] Cheng L Feng S Hu Y. Evidence-based nursing implementation in Mainland China: a scoping review. Nurs Outlook. 2017;65:27–35. 27665492 10.1016/j.outlook.2016.07.016

[26] Scott K McSherry R. Evidence-based nursing: clarifying the concepts for nurses in practice. J Clin Nurs. 2009;18:1085–95. 19077021 10.1111/j.1365-2702.2008.02588.x

